# Interplay between the gold nanoparticle sub-cellular localization, size, and the photon energy for radiosensitization

**DOI:** 10.1038/s41598-017-13736-y

**Published:** 2017-10-16

**Authors:** Eli Lechtman, Jean-Philippe Pignol

**Affiliations:** 1 Department of Medical Biophysics, University of Toronto at Sunnybrook Health Sciences Centre, Ontario, Canada; 2 Department of Radiation Oncology, University of Toronto, Ontario, Canada; 3 Department of Radiation Oncology, Erasmus MC Cancer Centre, Rotterdam, The Netherlands; 4 Department of Radiation, Science & Technology, TU Delft, Delft, The Netherlands

## Abstract

There are large variations in the reported efficiency of gold nanoparticle (GNP) radiosensitization. We have previously reported on a predictive model, which accounts for the detailed Auger and photoelectron tracks to calculate the cell survival probability. After validating our model using PC-3 cells incubated with 2 mg/ml of 30 nm GNPs and irradiated with 100 kVp or 300 kVp beams, we evaluated the interplay between photon energy, GNP size (1.9 and 100 nm) and sub-cellular localization. Experiments were in excellent agreement with the model. In predictive modeling, using a 100 kVp source and 1.9 nm nanoparticles, GNP localization had a significant impact on cell survival. A sensitizer enhancement ratio of 1.34 was achieved when GNPs were localized outside the cells, increasing to 2.56 when GNPs were also distributed in the cytoplasm and nucleus. Using a 300 kVp source, which emits photons mainly above the gold K-edge, the dependence on GNP localization and size was barely detectable, since long ranged electrons dominate the energy deposition. In summary, achieving intracellular uptake with targeted-GNPs can significantly enhance radiosensitization for photon energies below the gold K-edge, where Auger electrons contribute significantly to the local energy deposition. For higher energies, this is much less important.

## Introduction

Gold nanoparticles (GNPs) have been proposed to physically enhance radiation therapy^1–7^. The mechanism of radiosensitization involves three different steps: i/- the selective accumulation of GNPs in the tumor at close proximity to the cancer cell nucleus target; ii/- an increased photoelectric absorption of low-energy photons within gold atoms at the tumor site; iii/- the release of low-energy Auger and photoelectrons from GNPs, and the interaction of these secondary electrons with sensitive cellular targets^8^. GNP radiosensitization introduces a major modification to the pattern of energy deposition, leading to a more heterogeneous microscopic dose distribution, and resulting in electrons with very short ranges, increased linear energy transfer (LET), and a larger relative biological effectiveness (RBE).

The efficacy of GNP radiosensitization has been demonstrated *in vitro* in DNA plasmids and cell models^1–5^, and *in vivo* in mice model^6,7^. There is however very large variation in the reported RBE, which may be related to physical and pharmacological parameters including the primary photon energy spectrum, the GNP size, and the achievable tumor concentration and the sub-cellular localization^1–3^. The absorbed dose, which is the traditional metric correlated to a clinical effect, cannot adequately predict the effect of GNP radiosensitization on cell survival^9–13^. Our team has developed a radiobiological model called the gold (Au) nanoparticle radiosensitization predictive model (ARP). The ARP model was adapted from Kraft’s local effect model (LEM), which was designed to predict the increased RBE of heavy ion therapy^11–15^.

In the ARP model, photoelectric absorptions within GNPs are simulated, and detailed 3D nanoscale radiation transport and energy deposition of secondary radiation inside the cell nucleus are produced using Monte Carlo simulations^12^. Those elementary tracks are used to calculate the fractional probability of cell killing in 20 × 20 × 20 nm^3^ voxels of the cell nucleus using standard linear-quadratic radiosensitivity parameters. Finally, it calculates the cell survival probability by summing the fractional cell killing probabilities. This model incorporates information of the cancer cell geometry and intrinsic radiosensitivity, as well as the photon source spectrum, GNP size, concentration, and intracellular localization. In this article, after validating the ARP model with experimental clonogenic assays using various photons energies, we analyzed the subtle interplay between the photon source energy, GNP size and sub-cellular localization.

## Methods

### Experimental data validating the ARP model

*In vitro* experiments were carried out using the PC-3 human prostate adenocarcinoma cell line (American Type Culture Collection, Manassas, VA). Cells were maintained in RPMI 1640 with L-glutamine and sodium bicarbonate supplemented with 10% fetal bovine serum (Cellgro laboratories, Manassas, VA) and 5% penicillin with streptomycin (Invitrogen, Carlsbad, CA). Prior to experimentation, exponentially growing cells were seeded in 35 mm culture plates and grown to 80% confluence.

GNP colloids of 30 nm in diameter (Ted Pella Inc., Redding, CA) were PEGylated to prevent aggregation and then concentrated through centrifugation. Highly concentrated GNPs were re-suspended in cell culture media at gold concentrations of 2 mg/ml. Cell cultures were incubated with this mixture for 24 hours, then thoroughly washed with PBS to remove any nanoparticles not taken up in cells prior to irradiation and analysis. GNP cellular uptake was quantified using inductively coupled plasma mass spectroscopy (ICPMS) and GNP intracellular localization was visualized using transmission electron microscopy (TEM).

Irradiations were carried out on a clinical Gulmay D3300 (Chertsey, UK) x-ray therapy unit at energies of 300 kVp and 100 kVp using a 10 cm diameter cone collimator. Radiation was delivered from above the culture dishes penetrating 4 mm of cell culture media. Cultures of PC-3 cells with or without gold in the media for 24 hours were irradiated at 0, 1, 2, 4, or 8 Gy. Average cell survival was assessed using the clonogenic assay and normalized relative to the plating efficiency of controls receiving no radiation with and without GNPs. This was done to control for possible cytotoxic effects of GNPs. Survival as a function of dose for irradiations without GNPs was fit to the linear-quadratic cell survival model $S={e}^{-(\alpha D+\beta {D}^{2})}$, with the α and β parameters extracted using non-linear least-squares regression analysis in Matlab (MATLAB 7.14, The MathWorks Inc., Natick, MA, 2012).

### Monte Carlo simulation of GNP radiosensitization

The ARP model starts with the simulation of the 3D nanoscale energy deposition of photoelectric products escaping individual GNPs. To model this phenomenon while balancing computation efficiency, Monte Carlo simulations were performed using two different Monte Carlo Codes - MCNP-5 (Los Alamos National Laboratory) and PENELOPE 2008.1 (Barcelona, Catalonia)^16,17^. MCNP-5 includes powerful variance reduction tools and was used to generate the 100 kVp and 300 kVp photons phase space, as well as to calculate the rate of photoelectric absorption in GNPs after transport through 4 mm of cell culture media^12^. PENELOPE was used to simulate the interaction of photons with GNPs of 1.9, 30, and 100 nm diameters. PENELOPE was chosen due to the flexibility of its geometry package and customizable tallies. Furthermore, it offers detailed event-by-event electron transport through various mediums including tissue and gold down to 50 eV corresponding to electron ranges of about 2–4 nm. The individual tracks and nanoscale energy deposition of escaping electrons and photons were recorded using a customized tally^12^.

### The ARP model

The ARP model included a simplified three-compartment spherical cell model with a cytoplasm region, a radiosensitive nucleus region, and an extracellular region. PC-3 nucleus and cytoplasm dimensions were taken from previous studies^12,18^. GNP concentrations and distributions within these compartments were based on experimental data for model validation. They were then varied for the hypothetical cases testing the effect of GNP size, sub-cellular distribution and photon energy spectrum. Randomly selected secondary radiation tracks from a library of photons interactions within GNPs were applied to random locations within the sub-cellular compartments of a PC-3, and the energy deposition was scored within a matrix of 20 × 20 × 20 nm^3^ nucleus voxels. The background dose delivered by photons was then homogeneously distributed throughout the nucleus (see Supplementary Figure S1 for a schematic diagram of the ARP model).

Cell survival was determined by integrating the local dose response, or lethal event density, in each voxel over the nucleus volume as follow:

1 $$S={e}^{-\int v({D}_{local})dV}$$

The lethal event density was defined by a two component formulation based on the linear-quadratic cell survival model for local doses below a threshold value, *D*_*t*_, and a purely exponential survival model for local doses above the threshold to account for the limitation of the linear quadratic model at very high energy densities:

2 $$v({D}_{local})=\{\begin{array}{cc}\frac{\alpha {D}_{local}+\beta {D}_{local}^{2}}{{V}_{nucleus}} & when\,{D}_{local}\,\le \,{D}_{t}\\ v({D}_{t})+\frac{{S}_{max}({D}_{local}-{D}_{t})}{{V}_{nucleus}} & when\,{D}_{local}\, > {D}_{t}\end{array}$$

Where α and β represent the cell radiosensitivity parameters of the linear-quadratic model for low-LET photon radiation without GNPs present, D_local_ is the sum of the dose deposited from GNPs and the background radiation, and ${S}_{max}=\alpha +2\beta {D}_{t}$. The threshold dose for PC-3 cells has been previously empirically determined for the ARP model to be 23.9 Gy^12^.

### Metrics of comparison

For the validation of the ARP model, the mean inactivation dose (MID) was used as metric of comparison between experimental and theoretical data. The MID corresponds to the area under the cell survival curves. Sensitizer enhancement ratios (SER) were calculated by dividing the MID without GNPs by the MID with GNPs^1^.

### Data availability

The authors will make materials, data and associated protocols promptly available to readers upon request.

## Results

Table 1 presents the cellular dimensions obtained from previous publications, and summarizes the ICPMS results quantifying GNP cellular uptake concentrations. Because GNPs were not found in the nucleus on TEM imaging, the estimated concentration of gold in the cells was expressed per ml of cytoplasmic volume.

**Table 1:** Experimental PC-3 cell dimensions and GNP cellular uptake.

Cytoplasm radius ± SD (μm)	13.1 ± 2.5
Nucleus radius ± SD (μm)	8.2 ± 2.1
GNP size (nm)	30
GNP concentration in media (mg/ml)	2
GNPs per cell ± SD	2.27 × 10^4^ ± 1.47 × 10^4^
GNP concentration in cytoplasm (mg/ml)	0.84

**Figure 1:**
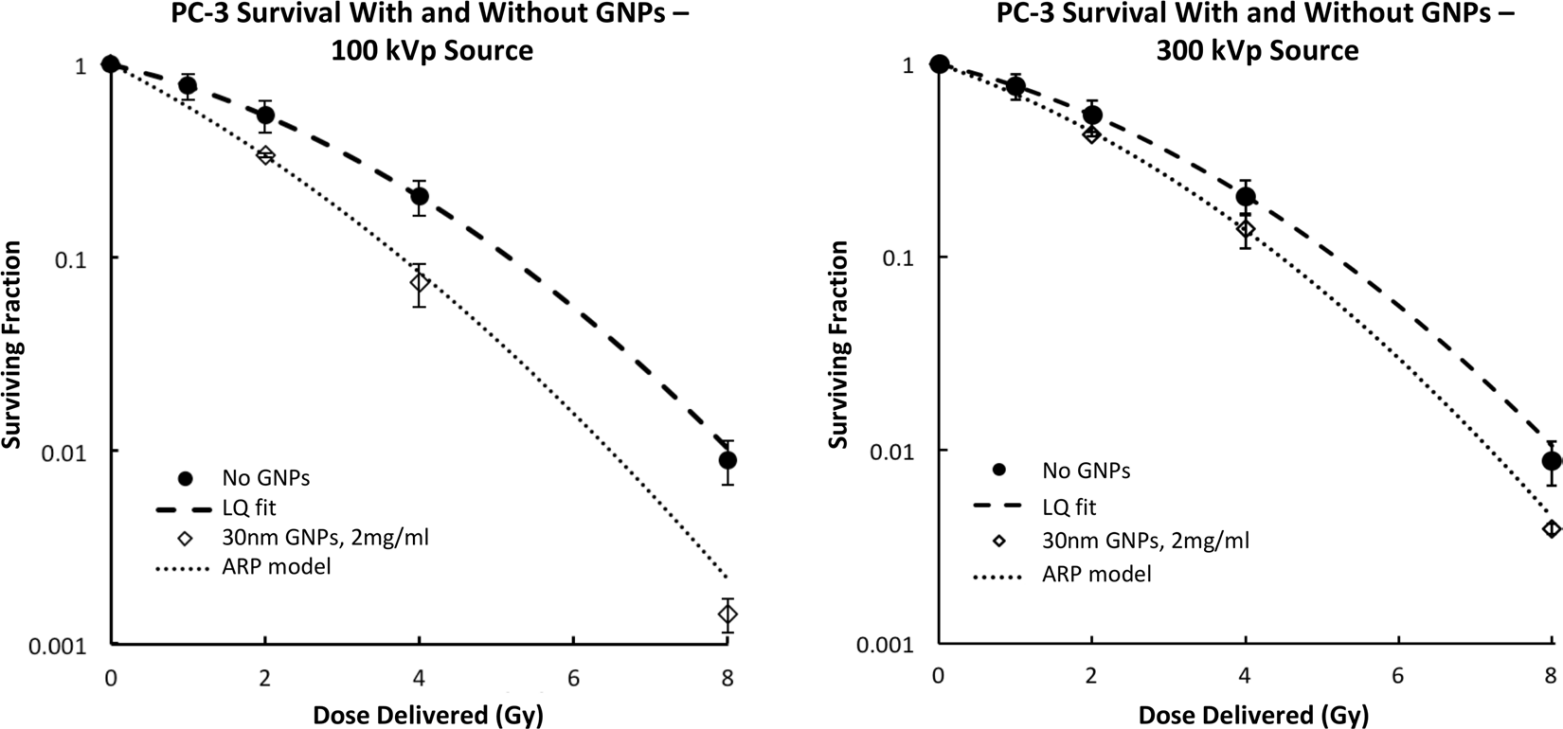
The ARP model shows close agreement with experimental cell survival for PC-3 cells incubated with 2 mg/ml of GNPs and irradiated using 100 kVp or 300 kVp photon energies. Cell survival with no GNPs was fit to the linear quadratic model (LQ) and depicted as well.

Figure 1 shows PC-3 experimental cell survival as a function of dose with and without 30 nm GNPs incubated at 2 mg/ml using the 100 kVp and the 300 kVp photon sources. There is a strong agreement with the ARP model predictions. Without GNPs, experimental PC-3 cell survival as a function of dose did not differ significantly between the 100 kVp and 300 kVp source, so the PC-3 cell sensitivity parameters were kept constant for both energies at α = 0.217 and β = 0.044 for the ARP survival model.

Using the 100 kVp photon source, Monte Carlo simulations estimated the rate of photoelectric absorption in a single 30 nm GNP to be 9.05 × 10^−3^ absorptions per 1 Gy delivered to the media. For a 300 kVp source the photoelectric absorption rate was estimated to be about 9 times less at 1.00 × 10^−3^ absorptions per 1 Gy.

**Figure 2:**
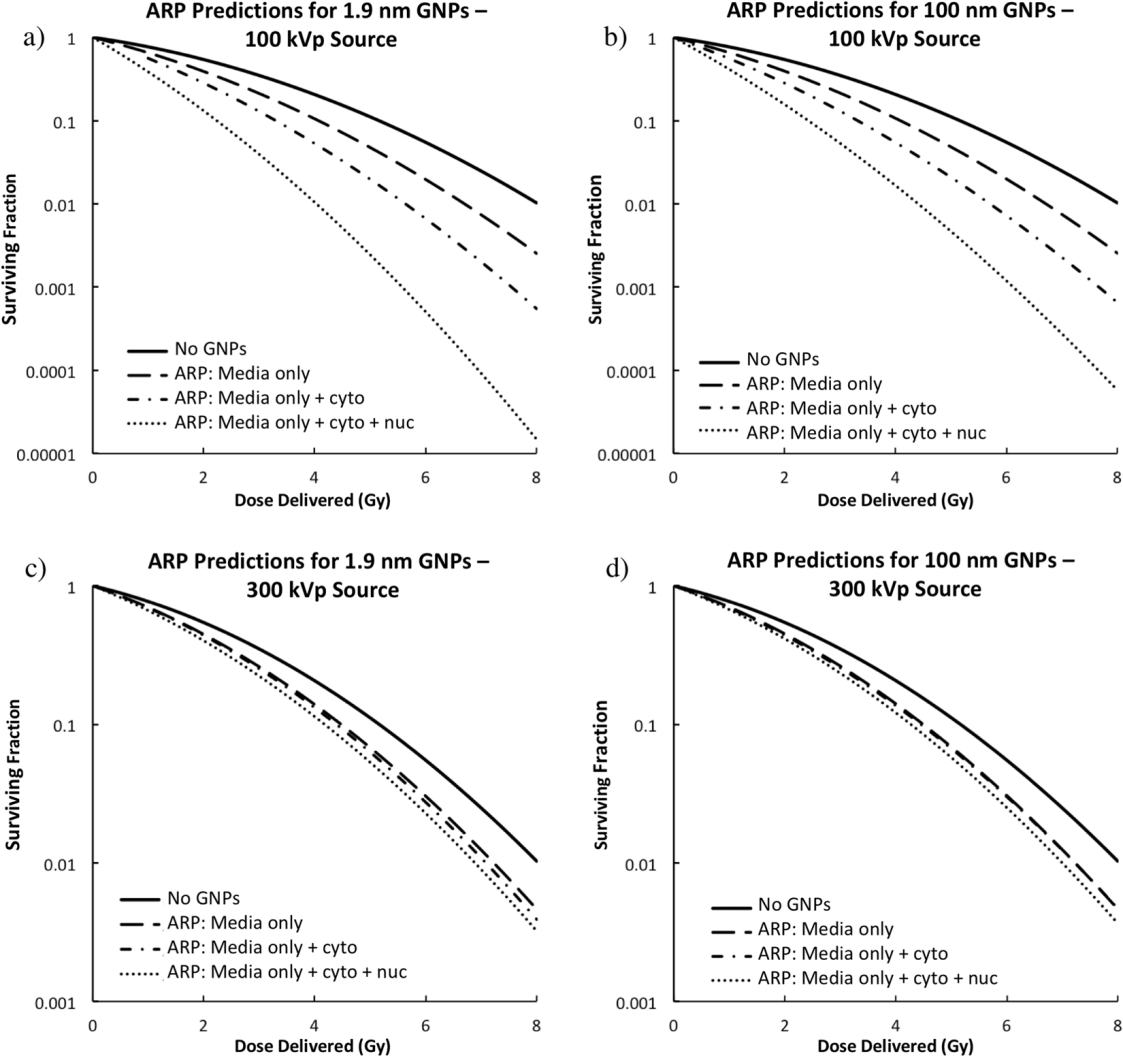
The predicted effects of varying GNP cellular and nuclear accumulation. When GNPs were simulated in a region, the concentration was set to 2 mg/ml. (**a**) ARP predictions using a 100 kVp source and 1.9 nm GNPs. (**b**) ARP predictions using a 100 kVp source and 100 nm GNPs. (**c**) ARP predictions using a 300 kVp source and 1.9 nm GNPs. (**d**) ARP predictions using a 300 kVp source and 100 nm GNPs.

Figure 2 shows predictive survival curves using either 100 or 300 kVp sources, varying sub-cellular distributions at a realistic concentration of 2 mg/ml, and for 1.9 nm or 100 nm GNPs. Three GNP localizations were considered for each case: 1) GNPs localized in the media only, 2) GNPs localized in the media and cell cytoplasm at the same concentration, 3) GNPs localized in the media, cytoplasm and nucleus. The effect of GNP localization was observed to be much more pronounced in the case of the 100 kVp source along with 1.9 nm GNPs. For this case, a SER of 1.34 was predicted when GNPs were localized outside the cells, increasing to 2.56 when GNPs were also distributed in the cytoplasm and nucleus. For the 300 kVp source, the dependence on GNP localization and size was barely detectable with a SER averaging about 1.2 for all cases. Varying the GNP size had a large influence on the cell survival when using the lower energy source and when nanoparticles were located in the cell nucleus. It had little or no influence for higher energy of when the GNP was in the cytoplasm or the media.

## Discussion

The ARP model was developed to account for the increased biological efficiency of short-ranged, high LET Auger electrons compared to long-ranged, low LET photoelectrons^12^. Similar to the LEM model, the ARP model exploits the unique and heterogeneous sub-cellular intra-nuclear energy deposition of high LET radiation^14,15^. The experimental cell survival values for 100 kVp and 300 kVp are in excellent agreement with the survival curves predicted by the ARP model for prostate PC-3 cancer cells. The two beam energies were specifically chosen because they yield photons with average energies of ≈33 keV and 100 keV respectively, meaning above or below the k-edge of gold (80.7 keV). Since the agreement is excellent, we assume that our simulations varying the sub-cellular localization or the size of the nanoparticles are reliable.

We hypothesized that cell survival would be highly dependent on the interplay between beam energy, and the size and localization of GNPs, and this is illustrated in Fig. 2. For the 300 kVp source, with a mean energy above the k-edge of gold, most of the primary photon energy is deposited by the longer-ranged photoelectrons, which do not have a significantly increased LET^12^. With this beam energy, the cell survival was not heavily influenced by GNP size or sub-cellular localization because the photoelectric products easily escape the nanoparticle and travel multiple cell lengths. At this energy, a modest SER of about 1.2 was observed, which may have a limited clinical significance.

On the other hand, using the 100 kVp source, with a mean energy below the gold k-edge, the energy is deposited more locally in the media by shorter-ranged electrons including high-LET Auger electrons, with maximum ranges of 1.5 μm, 0.1 μm, and 0.01 μm for transitions from the gold atom L, M, and N shells respectively^12^. Using the lower energy source, we found the cell survival to be highly dependent on the GNP size and localization. When GNPs were located too far from the nucleus, the short-range Auger electrons could not reach the DNA target. Furthermore, when GNPs were too large, the Auger electrons got trapped inside the large nanoparticle. In our simulations, the SER was maximized when using a low-energy source, along with small, nucleus-localized nanoparticles.

There have been several reports suggesting that besides the Auger cascade, other mechanisms could be at play to explain GNP radiosensitization^18–21^. However, if the goal is to maximize the Auger electrons production, the results presented in this article are central to designing a clinical strategy. If the intent is to use low-energy sources, such as miniature electronic x-ray or brachytherapy sources such as ^125^I or ^103^Pd, the radiosensitization could be dramatically enhanced by actively accumulating GNPs into the cell cytoplasm through conjugation with tumor targeting moieties^22,23^. Further enhancement could possibly be achieved through conjugation with nuclear localizing sequences^24,25^, but this would require GNPs small enough to pass through the ~5 nm nuclear pore complex, meaning that possibly much lower total gold concentrations would be achieved^26^. If the clinical application involves higher-energy sources, such as ^192^Ir used in high-dose-rate brachytherapy or external beam x-rays, the radiosensitization will mostly be linked to longer-ranged photoelectrons. In this case, GNP localization is less crucial and therefore a passive approach to GNP administration could be employed. GNPs would need to be loaded into tumors at relatively high concentrations of about 2 mg/ml, which could be achieved through direct intra-tumoral injection^27^.

The present modelization results suggest that for photon energies below the k-edge, the bulk gold concentration is not a good predictor for radiosensitization, and that knowledge of the intracellular concentration and localization is crucial. With this insight, the ARP model could explain some apparently contradictory reports of GNP radiosensitization. For example, Chithrani reported results of GNP radiosensitization using HeLa cells incubated with 50 nm GNPs at a media concentration of 0.0088 mg/ml^28^. Using a 105 kVp photon source, Chithrani observed cell survival comparable to our experimental results, despite the fact the incubated GNP concentration was 227 times lower than our study. This discrepancy could be explained by the fact that in both experiments similar intracellular gold concentrations were observed, between 6.3 μg and 7.6 μg, while the concentration in the media was indeed different. As we have shown for 100 kVp energies, the ARP model predicts that intracellular GNP concentration is a critical parameter.

One limitation of this work is that we used a highly simplified spherical cell geometry and assumed a uniform distribution of gold in sub-cellular compartments. This model functioned well to mimic the *in vitro* experiments, but the *in vivo* cellular and gross tumor morphology could be drastically different. GNPs can cluster into phagolysosomes and will be taken up at different concentrations by individual cancer cells^28^. The cell geometry evolves throughout the cell cycle, and can take on a spindle shape, with a more compact nucleus polarized to one side of the cell, closer to the extra-cellular space. Therefore, the DNA is a moving target, which could move closer or out of reach of the low-energy Auger electron spray arising from a nanoparticle^29^. Additional work is needed to evaluate the ARP model in realistic *in vivo* cellular geometry and with heterogeneous GNP distributions.

In this study, we utilized the ARP model to explore GNP radiosensitization with PC-3 prostate cancer cells and two clinical energies. However, the ARP model was designed as a general model that can be applied to various clinical scenarios, meaning using different beam energies and various cell radiosensitivity. We have theoretical results using ^125^I (average energy 28 keV) and ^192^Ir (average energy 395 keV) sources, which demonstrate the same trend of increasing SER with lower-energy sources, along with a stronger dependence on GNP localization. We have also conducted experiments with the SKBR-3 breast cancer cell line, with hence different α and β cell radiosensitivity values, and have obtained likewise excellent agreement with ARP model predictions.

To apply the ARP model to different biological conditions, experimental linear-quadratic α and β sensitivity parameters are required to accurately predict cell survival. This also opens the possibility to evaluate, at least in theory, additional clinical strategies that modify cell radiosensitivity. For example, the ARP model could provide insight into combining GNP radiosensitization with modifiers of the DNA repair pathway including lapatinib, hyperthermia which act through the inhibition of the BRCA homologous recombination DNA repair pathway^30,31^, or other specific clinical situations including hypoxia. On the other hand of the spectrum, this model could be use to evaluate strategies using Auger cascades radiosensitization^32^.

In summary, there is a significant interplay between GNP localization and size using low-energy sources below the k-edge. At those energies, the biological effect can be more than double. For higher energies, the GNP localization and size are much less important since the photo-electrons dominate the energy deposition.

## Electronic supplementary material

Supplementary figure 1
