# Supplementary material for: Interplay between the gold nanoparticle sub-cellular localization, size, and the photon energy for radiosensitization
Source: Sci Rep. 2017 Oct 16;7:13268. doi: 10.1038/s41598-017-13736-y (PMC5643548; doi:10.1038/s41598-017-13736-y)

INTERPLAY BETWEEN THE GOLD NANOPARTICLE SUB-CELLULAR LOCALIZATION, SIZE, AND THE PHOTON ENERGY FOR RADIOSENSITIZATION

Eli Lechtman MD PhD[1,2] and Jean-Philippe Pignol MD PhD[3,4*]

[1]Department of Medical Biophysics, University of Toronto at Sunnybrook Health Sciences Centre, Ontario, Canada

[2]Department of Radiation Oncology, University of Toronto, Ontario, Canada

[3]Department of Radiation Oncology, Erasmus MC Cancer Centre, Rotterdam, The Netherlands

[4]Department of Radiation, Science & Technology, TU Delft, Delft, The Netherlands

Supplementary Figure S1: A schematic representation of the ARP model. The 3D model cell consists of a cytoplasmic volume (in pink), and a nucleus volume (in blue). The cell depicted has a diameter of 13.1 microns. GNPs are randomly distributed in various compartments based on selected concentrations in the cytoplasm, nucleus or extracellular space. GNPs cannot be visualized at this scale. The cell is irradiated, and GNPs absorb then release photoelectric products. Shown are characteristic x-rays as dotted blue lines, and electrons in red. The dose deposited to tiny voxels within the nucleus is used to calculate the lethal event density, and this is integrated over the nucleus to determine cell survival.

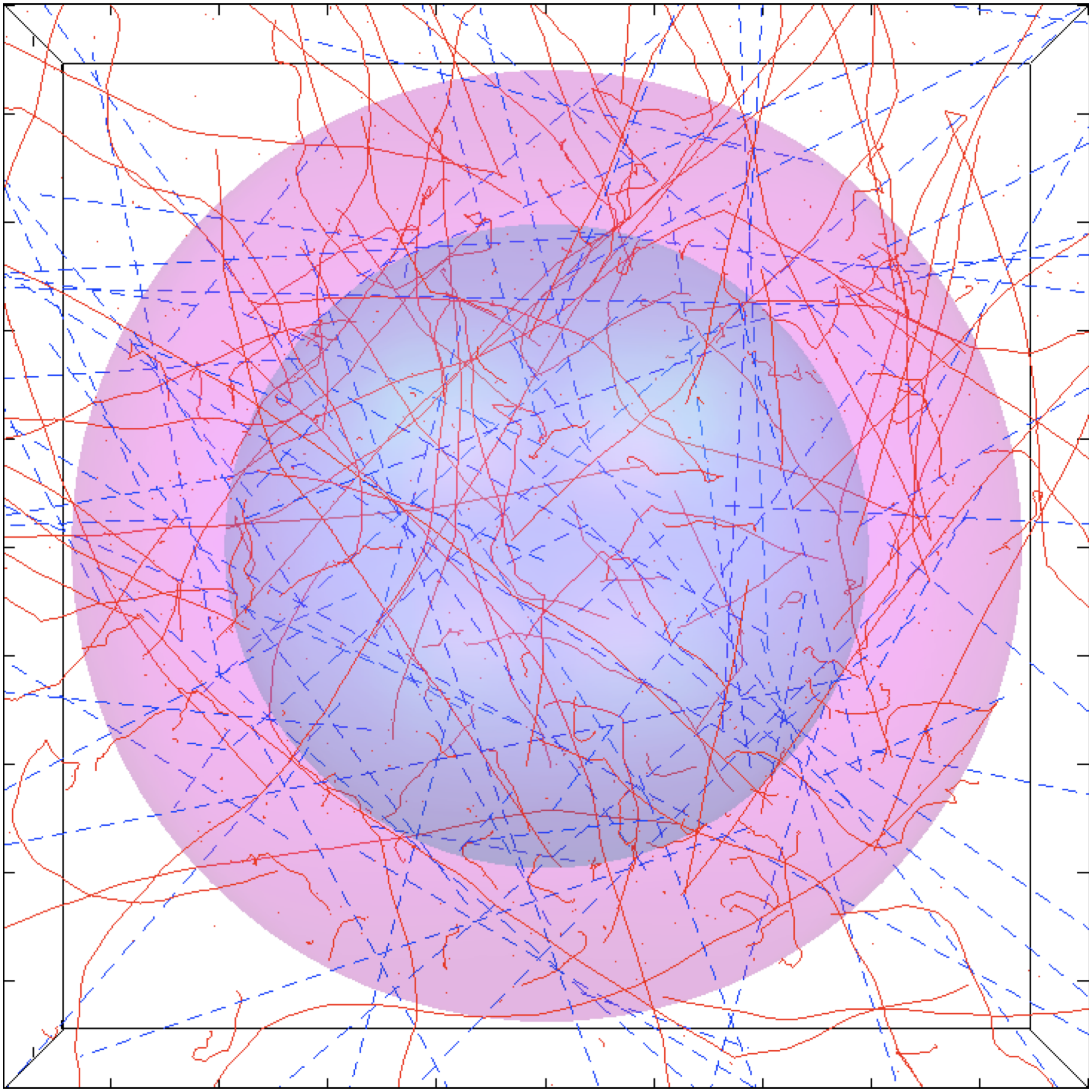

Supplement: Supplementary file 1 — Supplementary figure 1 [file 41598_2017_13736_MOESM1_ESM.pdf]
